# Structural Aspects of *E. coli* Type II Asparaginase in Complex with Its Secondary Product L-Glutamate

**DOI:** 10.3390/ijms23115942

**Published:** 2022-05-25

**Authors:** Maristella Maggi, Claudia Scotti

**Affiliations:** Unit of Immunology and General Pathology, Department of Molecular Medicine, University of Pavia, 27100 Pavia, Italy; maristella.maggi@unipv.it

**Keywords:** L-asparaginase, L-glutamate, catalysis

## Abstract

Bacterial L-asparaginases are amidohydrolases (EC 3.5.1.1) capable of deaminating L-asparagine and, with reduced efficiency, L-glutamine. Interest in the study of L-asparaginases is driven by their use as biodrugs for the treatment of acute lymphoblastic leukemia. Here, we report for the first time the description of the molecular structure of type II asparaginase from *Escherichia coli* in complex with its secondary product, L-glutamate. To obtain high-quality crystals, we took advantage of the N24S variant, which has structural and functional features similar to the wild-type enzyme, but improved stability, and which yields more ordered crystals. Analysis of the structure of the N24S-L–glutamate complex (N24S–GLU) and comparison with its apo and L-aspartate-bound form confirmed that the enzyme-reduced catalytic efficiency in the presence of L-glutamine is due to L-glutamine misfitting into the enzyme-binding pocket, which causes a local change in the catalytic center geometry. Moreover, a tight interaction between the two protomers that form the enzyme active site limits the capability of L-glutamine to fit into (and to exit from) the binding pocket of *E. coli* L-asparaginase, explaining why the enzyme has lower glutaminolytic activity compared to other enzymes of the same family, in particular the *Erwinia chrysanthemi* one.

## 1. Introduction

L-asparaginases are amidohydrolases (EC 3.5.1.1) found in almost all living organisms, from mammalian to plants, birds, yeast and bacteria [1,2,3]. The enzyme has two hydrolytic activities: the prevalent L-asparaginase and the secondary L-glutaminase. Prokaryotes are the main source of L-asparaginases with anti-cancer activity. Bacterial L-asparaginases are homotetrameric proteins of 140–150 kDa. Each subunit is built-up by nearly 330 amino acids and consists of two α/β domains connected by a ca 21 amino-acid-long random coil. A rare left-handed crossover in the N-domain is a distinguished feature of all bacterial L-asparaginases and it is highly evolutionarily conserved in all organisms [4]. Bacterial L-asparaginase active sites are constituted by a rigid part for ligand binding and a flexible one [2]. The latter is highly conserved in all L-asparaginases and controls access to the enzyme-binding pocket and the relocation of essential catalytic residues. The enzyme reaction consists of two nucleophilic attacks involving two highly conserved sets of amino acids residues, named catalytic triads, and several water molecules. The first triad, typically Thr-Lys-Asp, contains a nucleophilic residue (Thr), a Lys that acts as a general base, and an acidic group (Asp) [5,6]. Residues belonging to the enzyme second triad (Thr-Tyr-Glu) are mainly involved in substrate binding and/or product release. The enzyme reaction starts with a nucleophilic attack on the substrate amide C-atom, that forms the enzyme acyl intermediate with the nucleophilic threonine of the first triad (T12 in *E. coli* asparaginase, EcAII [7]). In type II bacterial asparaginases, the presence of a peculiar and highly conserved oxyanion hole contributes to the stabilization of the acyl intermediate and to the formation of the tetrahedral intermediate, which is then solved by a second nucleophilic attack [2]. Here, a water molecule plays the main role in product release (w II according to Maggi et al. [8], w2 according to Lubkowski et al. [9,10]).

The catalytic mechanism of asparaginases has mainly been described for the enzyme asparaginolytic activity. The general scheme of reaction can also be applied to the process of L-glutamine deamination, but it is well known that bacterial asparaginases have much less catalytic efficiency in the presence of L-glutamine than of L-asparagine [11,12]. Indeed, type II L-asparaginases from *E. coli* and *E. chrysanthemi* (ErAII), the only two L-asparaginases with clinical interest for the treatment of acute lymphoblastic leukemia [13], are endowed with glutaminase activity that is 0.065% [14] and 1.5% [15], respectively, of L-asparaginase activity. The molecular reasons for the different behavior have not been fully elucidated so far, especially for the *E. coli* enzyme.

According to the Protein Data Bank accessed at the time of writing and to the state-of-the-art review recently published by Lubkowski [16], the structural features of *E. coli* L-asparaginase in complex with the product L-glutamate (L-GLU) are still undefined. Here, we report it for the first time, disclosing some details of the interactions between the well-characterized catalytic elements of the enzyme and its secondary product. To obtain high-quality crystals, we took advantage of the highly stable N24S L-asparaginase, which shares identical catalytic and structural properties with the wild-type enzyme, but has largely improved stability and forms highly ordered crystals [14].

## 2. Results

### 2.1. Overall Apo-N24S Structure

The asymmetric unit (ASU) of apo-N24S, solved at 1.70 Å resolution, comprised 4 monomers, named A, B, C, and D. Chains A/B and C/D are associated into two independent intimate dimers. The biological unit was rebuilt using the crystallographic C2 symmetry operators. None of the protomers contained a complete electron density trace in the region corresponding to the extremely mobile active site flexible loop (ASFL). Protomer A missed residues 15–30, protomer B 16–31, protomer C 16–32, and protomer D, the most complete, 15–25. In all cases, the active site was free of ligands and the rebuilt ASFL was in a position compatible to the one of the enzyme in its open conformation (compare to 7P9C [8]). The overall fold of apo-N24S resembled the one of previously described *E. coli* type II L-asparaginases (e.g., [4,8,14,17]).

### 2.2. Overall N24S–GLU Structure

**Figure 1:**
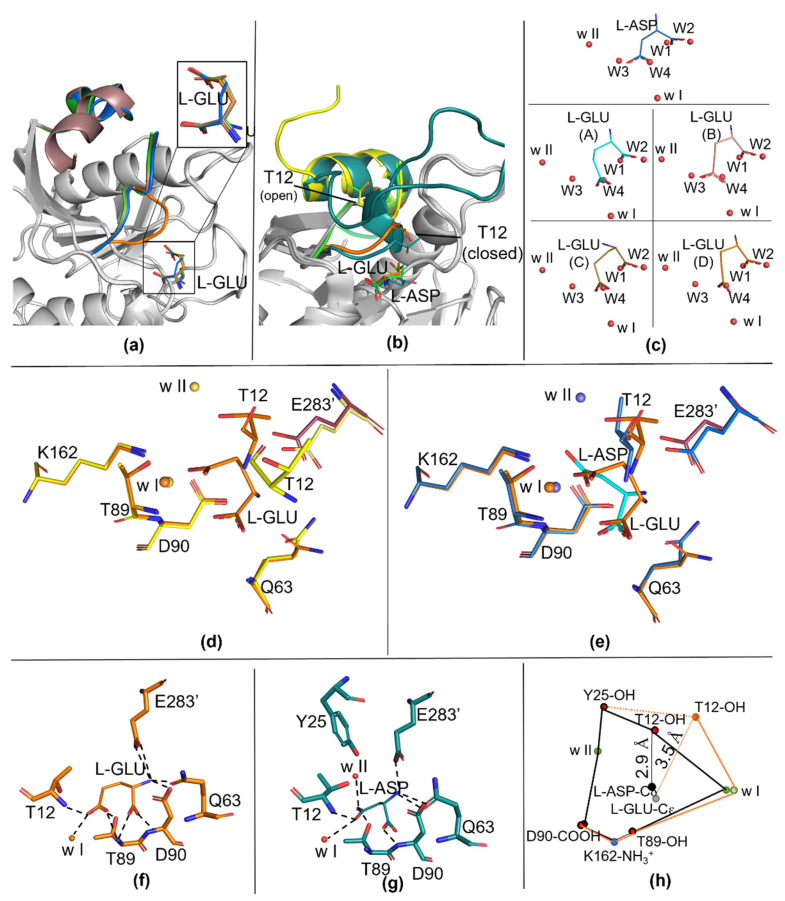
Details of the catalytic site of apo-N24S (7R59) and N24S–GLU (7R5Q). (**a**) N24S–GLU protomers superposition with details of L-GLU conformation (top right rectangle). The active site flexible loop (ASFL) is represented as a cartoon and colored in blue for protomer A, dark pink for protomer B, green for protomer C and orange for protomer D. (**b**) Details on the ASFL organization in apo-N24S (PDB ID: 7R57, yellow), in N24S–ASP (PDB ID: 5MQ5, dark green) and in N24S–GLU (PDB ID: 7R5Q: green, protomer C in its open conformation; orange, protomer D in its closed conformation). The products (L-ASP and L-GLU) and the catalytic T12 residue are represented as sticks, ASFL is represented as a cartoon. (**c**) Water molecules displacement in the substrate-binding pocket. L-ASP and L-GLU are represented as sticks. Water molecules are represented as red spheres. Waters present in the apo-N24S structure and replaced by the product in the ligand-bound structures are named W1 to W4; highly conserved, catalytic waters are named w I and w II. Top panel: ligand in N24S–ASP. Bottom panel: ligand in the four protomers of N24S–GLU. (**d**,**e**) Comparison of catalytic centers: in (**d**), apo-N24S (yellow) and N24S–GLU (orange), in (**e**), N24S–ASP (dark green) and N24S–GLU. Products and catalytic residues are represented as sticks, waters are represented as spheres. (**f**,**g**) L-GLU and L-ASP polar contacts. (**h**) Distances of catalytically relevant chemical groups in N24S–ASP (black lines) versus N24S–GLU (orange lines). The connection between Y25 and T12 in N24S–GLU is represented as a dotted line, as Y25 could not be modelled in the N24S–GLU structure.

The ASU of the N24S–GLU complex, solved at 1.90 Å resolution, was similar to the one of apo-N24S: it comprised the 4 A, B, C, and D monomers, with chains A/C and B/D forming the two independent intimate dimers and the biological unit rebuilt using the crystallographic C2 symmetry operators. Even here, none of the protomers in the ASU contained a complete electron density trace for the ASFL. Protomer A missed clear electron density signal for residues 16–31, protomer B 16–35, protomer C 16–30 and protomer D 15–37. All the active sites contained clear electron density for the L-GLU ligand (Supplementary Figure S1). Positions of L-GLU atoms were superposable for all protomers except protomer B, in which the L-GLU Cβ was 1.7 Å shifted with respect to the corresponding atom of the other ligands (Figure 1a). For protomers A, B and C, the position of the visible part of the ASFL is compatible with the position of the enzyme in its open conformation (see, for example, PDB ID 7P9C and 7R57), while, in the case of protomer D, it is compatible with its closed conformation (Figure 1b) (see, for example, PDB ID 5MQ5, N24S–ASP complex). In Table 1, collection and refinement statistics for both structures are reported.

### 2.3. Ligand and Active Site Residues Displacement

Analysis of the solvent content inside the apo-N24S-binding pocket confirms the presence of the four highly conserved water molecules (named W1–W4) that are known to coordinate and interact with the rigid part of the substrate-binding pocket [8,9]. As previously observed for the wild-type apo-enzyme, in fact, the position of each of the four water molecules exactly coincides with one of the oxygen atoms of the two carboxylic groups of the bound L-ASP ligand (Figure 1c). In the case of our structure, in all four N24S–GLU protomers, the oxygen atoms of the Cα carboxylic group of L-GLU are, in fact, located in the positions normally occupied by waters W1 and W2 in the apo-N24S structure. However, because of the extra CH_2_ group (Cγ) in its side chain compared to the one of L-ASP, the O atoms of the Cδ carboxylic group of L-GLU do not coincide either with the position of the apo-N24S W3 and W4 nor of the L-ASP Cγ carboxylic group O atoms (Figure 1c). As a consequence of the steric hindrance induced by L-GLU “misplacement” in the binding pocket, the catalytic residues T12, Q63 and E283 are shifted from the position usually observed in both the apo- and N24S–ASP structures (Figure 1d,e, respectively). Additionally, w II, which is involved in the second step of the catalysis that resolves the enzyme tetrahedral and releases the deaminated product, is absent in three of the four protomers, being present only in protomer B, where L-GLU has a conformation more similar to L-ASP (Figure 1c).

**Figure 2:**
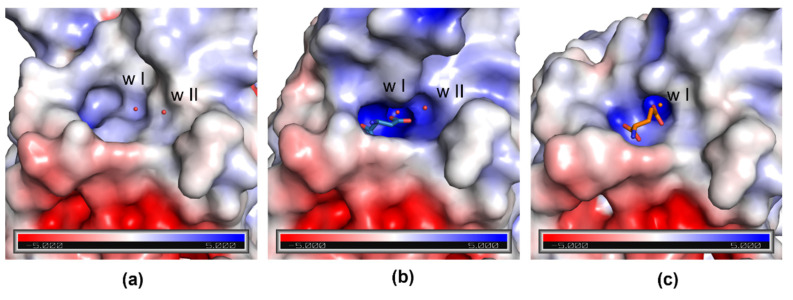
Electrostatic potential surface of apo-N24S (**a**), N24S–ASP (**b**), and N24S–GLU (protomer D) (**c**) binding sites. Catalytic waters w I and w II are represented as red spheres, L-ASP and L-GLU as sticks. In N24S–ASP (**b**), part of the ASFL is not shown to make the binding pocket visible.

Comparison of the electrostatic properties of the protein surface shows that an oxyanion hole, which is essential for the stabilization of the tetrahedral intermediate, is visible in the closed conformation of N24S–GLU protomer D (Figure 2c). Like in the enzyme wild type (PDB ID: 7P9C), the oxyanion hole is absent in apo-N24S (Figure 2a) and strongly evident in N24S–ASP (Figure 2b). Water w I is embedded in the internal part of the hole and is present in all three structures; water w II, instead, has a marginal position in the oxyanion hole and is absent in N24S–GLU.

## 3. Discussion

In this paper, we describe for the first time the structure of *E. coli* asparaginase (N24S mutant) in complex with its secondary product, L-GLU. The high-resolution structure (1.9 Å) provides essential information about the molecular aspects that characterize the interactions between the enzyme catalytic center and its ligand. In particular, the presence of the structurally and catalytically relevant active site flexible loop, both in its open and closed conformation, even if only partially modelled, allows insights on the capability of the enzyme to acquire a catalytically competent conformation in the presence of the L-GLN substrate. According to the collected data, the ASFL closure is less efficient in the presence of L-GLU, very likely because of the steric hindrance deriving from the extra methyl group of the side chain compared to L-ASP. Functional characterization of N24S, in fact, confirms a reduced efficiency in L-GLN binding with a K_m_ 138-fold higher than in the presence of L-ASN (4.14 vs. 0.03 mM, respectively [14]). As a further confirmation, superposition of N24S–GLU- and apo-N24S-binding sites shows that the position of L-GLU oxygen atoms do not perfectly coincide with that of water molecules involved in stabilizing contacts with key binding site residues (Figure 1c). In the structure of N24S–GLU protomer D, in which the ASFL is in a conformation compatible with its closed position, the placement of L-GLU inside the binding pocket causes the shifting of catalytically relevant residues from their canonical position, causing an altered chemical geometry in the catalytic center (Figure 2) that, in turn, can explain the less efficient catalysis. Moreover, the difficult placement of L-GLU in the binding pocket causes a local increase in hydrophobicity in the active site and impairs proper placement of the primary catalytic T12 and of water w II, which is involved in the second step of the catalysis and in the release of the product. Hence, L-GLU conformation might also hamper its release after the first step of catalysis, further reducing the enzyme turnover efficiency compared to L-ASN. Indeed, in the presence of L-GLN as a substrate, N24S turnover number is approximately 110-fold lower than in the presence of L-ASN as a substrate (k_cat_, 0.53 vs. 58.81 sec^−1^, respectively [14]).

**Figure 3:**
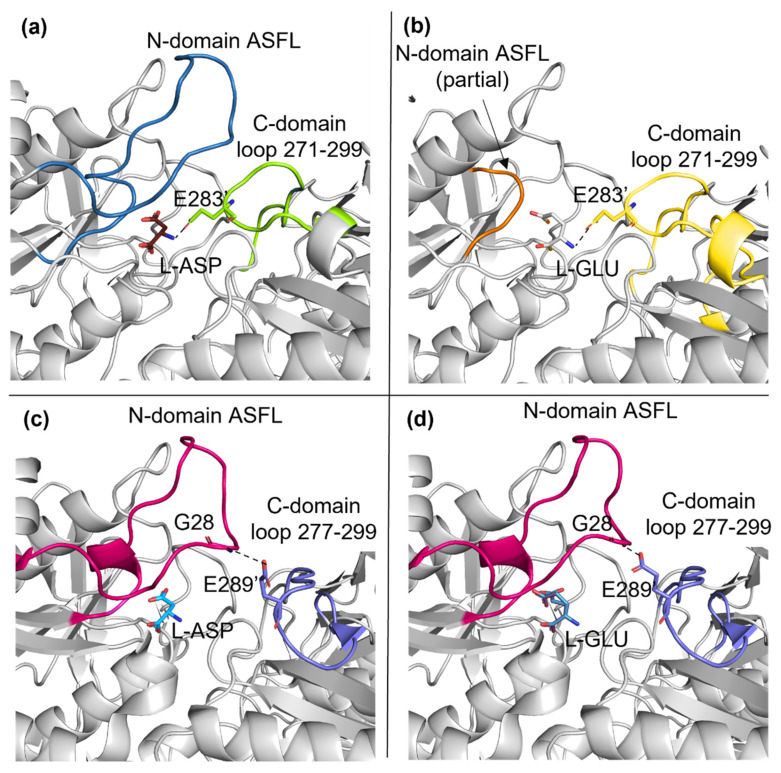
Comparison of EcAII (N24S mutant) and ErAII. (**a**) N24S–ASP (5MQ5) and (**b**) N24S–GLU (7R5Q) active site at the interface of the intimate dimer. ASFL and the C-domain loop are represented as a cartoon and colored in dark blue and light green (**a**), respectively, and in orange and light yellow (**b**), respectively. L–ASP, L–GLU and E283 are represented as sticks. (**c**) ErAII–ASP (5F52) and (**d**) ErAII–GLU (5HW0) active site at the interface of the intimate dimer. ASFL and the C–domain loop are represented as cartoons and colored in magenta and purple, respectively. L–ASP, L–GLU, G28 and E289 are represented as sticks.

The structure of *E. chrysanthemi* asparaginase (ErAII) in complex with L-GLU was previously released [18] and similar local changes were described comparing the structures of ErAII in complex with either L-ASP or L-GLU. Nevertheless, ErAII is reported to be more efficient than EcAII in catalyzing L-glutamine deamination. The improved catalytic efficiency depends on ErAII higher affinity and specific activity towards L-GLN with respect to EcAII (10- and 5-fold higher, respectively [15]). Structurally, the main difference between ErAII and EcAII active sites is the positioning of a key loop located in the C-domain of the companion monomer in the functional intimate dimer (residues 277–299 in ErAII and 271–299 in EcAII). In EcAII, the C-domain loop is located in proximity of the binding site and acquires direct contacts with the substrate (either L-ASP or L-GLU) N-group through E283 (Figure 3a,b), which is also involved in contacts with the catalytic residues Y25 (in the closed conformation) and Q63 (in both open and closed conformations). In ErAII, the C-domain loop establishes contacts only with G28, a residue belonging to the ASFL and not involved in the catalytic process. Such a tight contact between the two monomers in EcAII intimate dimers (present also in the enzyme apo-structure) makes the size of the opening, useful for substrate access and product exiting from the active site, quite narrow. This represents the structural basis of the enzyme-reduced glutaminolytic turnover number and can explain the reduced capability of EcAII to efficiently deaminate L-glutamine as a substrate.

In conclusion, the newly described EcAII N24S–GLU structure provided experimental evidence of the so far only hypothesized re-organization of the enzyme catalytic center in the presence of its secondary substrate and poses the basis for further rational engineering of the drug to modulate its secondary glutaminase activity.

## 4. Materials and Methods

### 4.1. Protein Production and Crystallization

Recombinant N24S was produced and purified as previously described [14]. Protein pure to homogeneity was dialyzed versus 30 mM Tris–HCl buffer pH 8.0 and used for protein crystallization at 4 mg/mL. Crystals were grown at 21 °C in a sitting drop vapor diffusion setting, mixing 1 μL protein with 1 μL mother liquor. The crystallization condition for both apo–N24S and N24S–GLU was obtained by optimization of condition MD1-02 #19 of the sparse-matrix screening from Molecular Dimensions (MD1–03) and contained 100 mM HEPES (4-(2-hydroxyethyl)-1-piperazineethanesulfonic acid) pH 8.0, 5% *w*/*v* PEG 8000, 4% *v*/*v* ethylene glycol for the apo crystals, and 100 mM HEPES, pH 7.5, 8% *w*/*v* PEG 8000, 6% *v*/*v* ethylene glycol for the L–GLU–crystals. Upon cryo–mounting on nylon loops, crystals to be analyzed by X-ray were soaked in 500 μM L–glutamic acid (L-GLU) dissolved in mother liquor for 10 min to obtain N24S–GLU complex crystals.

### 4.2. Data Collection and Refinement

X-ray diffraction data were collected at the Europeans Synchrotron Facility (ESRF, Grenoble, France) at beamline ID–29 (apo–N24S) and ID30A–1 (N24S–GLU). The data were processed using XDS and AIMLESS. The structure was solved by molecular replacement with Phaser and by using a single monomer derived from the deposited EcAII structure (3ECA) as a probe. The structure was refined alternating cycles of refinement (Phenix, real space, reciprocal space, individual B factors and occupancy) and manual rebuilding based on electron density maps (Coot). In the case of N24S–GLU, the ASU was rebuilt to contain the two intimate dimers and submitted to the ACHESYM server. The final structures were deposited into the Protein Data Bank (PDB) with IDs 7R57 (apo–N24S) and 7RQ5 (N24S–GLU). Images were produced by Pymol. Electrostatic potential surface (ESP) was calculated using the Adaptive Poisson-Boltzmann Solver (APBS) method plug-in for Pymol.

## Figures and Tables

**Table 1:** Data collection and refinement statistics.

Structure	PDB ID	Property	Value
apo-N24S	7R57	**Data collection**	
		Space group	C2
		Cell constants (a, b, c)	152.14 Å, 62.44 Å, 143.41 Å
		Cell constants (α, β, γ)	90.00°, 118.19°, 90.00°
		Resolution	47.09–1.40 Å
		R_merge_	0.05
		I/σI	1.4
		Completeness in resolution range	98.5%
		Multiplicity	2.8
		**Refinement**	
		Resolution range	47.09–1.70 Å
		No. of reflections	128,815
		R_work_/R_free_	0.17/0.19
		R.m.s. bond length	0.01 Å
		R.m.s. bond angles	1.02°
N24S–GLU	7R5Q	**Data collection**	
		Space group	C2
		Cell constants (a, b, c)	151.03 Å, 61.95 Å, 142.56 Å
		Cell constants (α, β, γ)	90.00°, 118.31°, 90.00°
		Resolution	47.81–1.57 Å
		R_merge_	0.07
		I/σI	1.26
		Completeness in resolution range	99.3
		Multiplicity	2.7
		**Refinement**	
		Resolution range	47.811–1.90 Å
		No. of reflections	90,818
		R_work_/R_free_	0.17/0.20
		R.m.s. bond length	0.0079 Å
		R.m.s. bond angles	0.93°

## Data Availability

The N24S structure in its apo and L-GLU-bound form is available in the Protein Structure Database under IDs 7R57 and 7R5Q, respectively.

## Supplementary Materials

The following supporting information can be downloaded at: https://www.mdpi.com/article/10.3390/ijms23115942/s1.

## References

[1] Zuo S., Zhang T., Jiang B., Mu W. (2014). Recent Research Progress on Microbial L-Asparaginases. Appl. Microbiol. Biotechnol..

[2] Michalska K., Jaskolski M. (2006). Structural Aspects of L-Asparaginases, Their Friends and Relations. Acta Biochim. Pol..

[3] Maggi M., Chiarelli L.R., Valentini G., Scotti C. (2015). Engineering of *Helicobacter Pylori* L-Asparaginase: Characterization of Two Functionally Distinct Groups of Mutants. PLoS ONE.

[4] Swain A.L., Jaskólski M., Housset D., Rao J.K., Wlodawer A. (1993). Crystal Structure of *Escherichia Coli* L-Asparaginase, an Enzyme Used in Cancer Therapy. Proc. Natl. Acad. Sci. USA.

[5] Nomme J., Su Y., Lavie A. (2014). Elucidation of the Specific Function of the Conserved Threonine Triad Responsible for Human L-Asparaginase Autocleavage and Substrate Hydrolysis. J. Mol. Biol..

[6] Maggi M., Chiarelli L.R., Valentini G., Scotti C. (2015). Tackling Critical Catalytic Residues in *Helicobacter Pylori* L-Asparaginase. Biomolecules.

[7] Palm G.J., Lubkowski J., Derst C., Schleper S., Röhm K.-H., Wlodawer A. (1996). A Covalently Bound Catalytic Intermediate in *Escherichia Coli* Asparaginase: Crystal Structure of a Thr-89-Val Mutant. FEBS Lett..

[8] Maggi M., Meli M., Colombo G., Scotti C. (2021). Revealing *Escherichia Coli* Type II L-Asparaginase Active Site Flexible Loop in Its Open, Ligand-Free Conformation. Sci. Rep..

[9] Lubkowski J., Wlodawer A. (2019). Geometric Considerations Support the Double-Displacement Catalytic Mechanism of l-Asparaginase. Protein Sci..

[10] Lubkowski J., Vanegas J., Chan W.K., Lorenzi P.L., Weinstein J.N., Sukharev S., Fushman D., Rempe S., Anishkin A., Wlodawer A. (2020). Mechanism of Catalysis by l-Asparaginase. Biochemistry.

[11] Strzelczyk P., Zhang D., Dyba M., Wlodawer A., Lubkowski J. (2020). Generalized Enzymatic Mechanism of Catalysis by Tetrameric L-Asparaginases from Mesophilic Bacteria. Sci. Rep..

[12] Covini D., Maggi M., Tardito S., Bussolati O., Chiarelli L. R., Pasquetto M.V., Vecchia L., Valentini G., Scotti C. (2014). Expanding Targets for a Metabolic Therapy of Cancer: L-Asparaginase. Top. Anti-Cancer Res..

[13] Maggi M., Scotti C. (2019). Enzymes in Metabolic Anticancer Therapy. Adv. Exp. Med. Biol..

[14] Maggi M., Mittelman S.D., Parmentier J.H., Colombo G., Meli M., Whitmire J.M., Merrell D.S., Whitelegge J., Scotti C. (2017). A Protease-Resistant *Escherichia Coli* Asparaginase with Outstanding Stability and Enhanced Anti-Leukaemic Activity in Vitro. Sci. Rep..

[15] Nguyen H.A., Su Y., Lavie A. (2016). Design and Characterization of *Erwinia Chrysanthemi* L-Asparaginase Variants with Diminished l-Glutaminase Activity. J. Biol. Chem..

[16] Lubkowski J., Wlodawer A. (2021). Structural and Biochemical Properties of L-Asparaginase. FEBS J..

[17] Sanches M., Barbosa J.A., de Oliveira R.T., Abrahão Neto J., Polikarpov I. (2003). Structural Comparison of *Escherichia coli* L-Asparaginase in Two Monoclinic Space Groups. Acta Crystallogr. D Biol. Crystallogr..

[18] Aghaiypour K., Wlodawer A., Lubkowski J. (2001). Structural Basis for the Activity and Substrate Specificity of *Erwinia Chrysanthemi* L-Asparaginase. Biochemistry.

