# Supplementary material for: Structural Aspects of E. coli Type II Asparaginase in Complex with Its Secondary Product L-Glutamate
Source: Int J Mol Sci. 2022 May 25;23(11):5942. doi: 10.3390/ijms23115942 (PMC9180372; doi:10.3390/ijms23115942)
Supplement: Supplementary file 1 [file ijms-23-05942-s001.zip › ijms-1716149-supplementary.pdf]

Supplementary Material

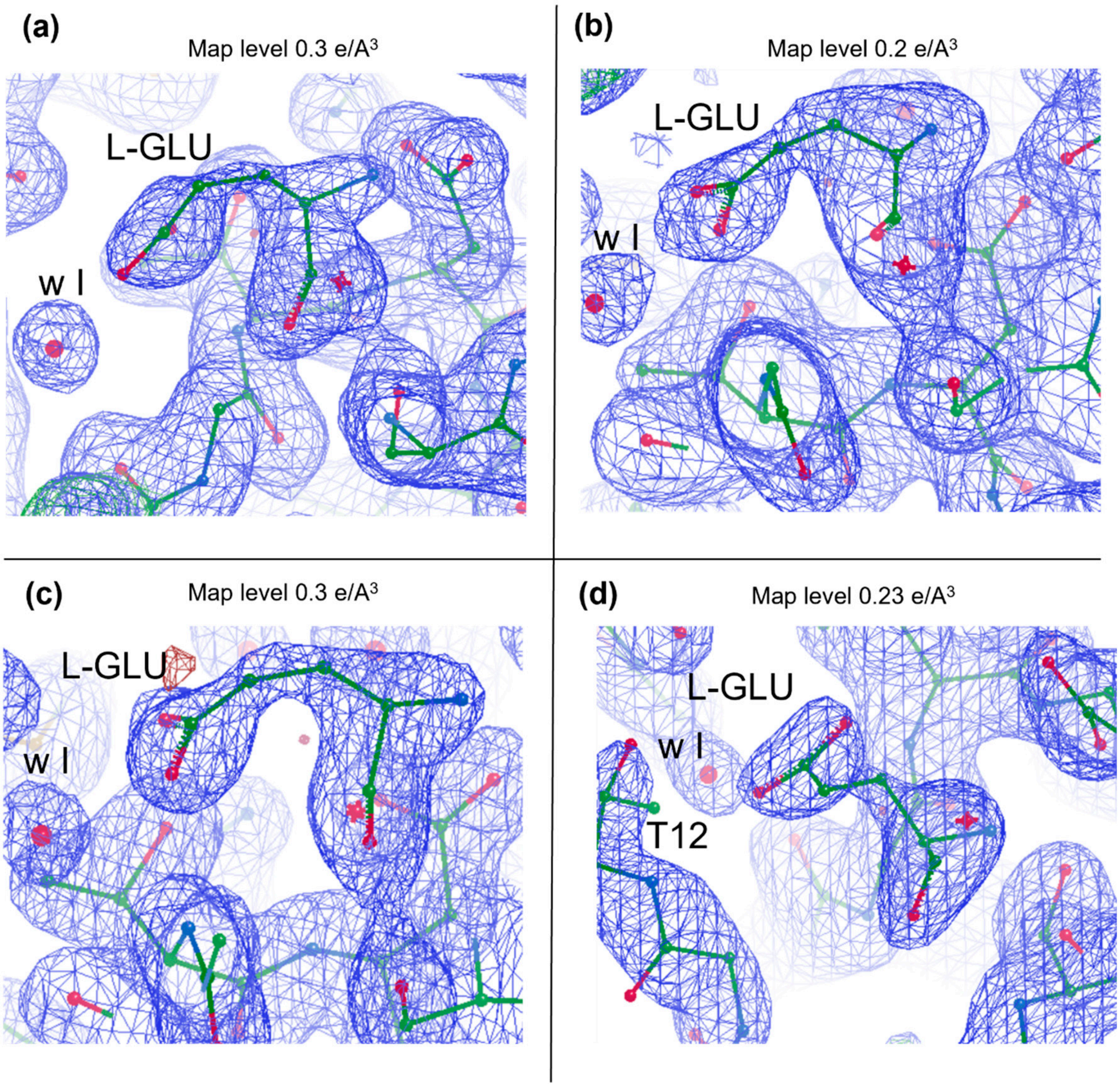

Figure S1. N24S-GLU (PDB ID: 7R5Q) electron density map of the 4 L-GLU molecules in each of the ASU protomers. (**a**) protomer A, (**b**) protomer B, (**c**) protomer C, (**d**) protomer D. L-GLU, catalytic water I (w I) and T12 (modelled only in protomer D) are labelled. Orientation of the maps was chosen to favour the visualization of the L-GLU product electron density.
